# Structural characterization of the apo form and NADH binary complex of human lactate dehydrogenase

**DOI:** 10.1107/S1399004714005422

**Published:** 2014-04-30

**Authors:** Sally Dempster, Stephen Harper, John E. Moses, Ingrid Dreveny

**Affiliations:** a School of Chemistry, University of Nottingham, Nottingham NG7 2RD, England; b School of Pharmacy, Centre for Biomolecular Sciences, University of Nottingham, Nottingham NG7 2RD, England

**Keywords:** lactate dehydrogenase, glycolysis, conformational change, ligand soaking, apo structure

## Abstract

Lactate dehydrogenase A (LDH-A) is a key enzyme in anaerobic respiration that is predominantly found in skeletal muscle and catalyses the reversible conversion of pyruvate to lactate in the presence of NADH. LDH-A is overexpressed in many tumours and has therefore emerged as an attractive target for anticancer drug discovery. Crystal structures of human LDH-A in the presence of inhibitors have been described, but currently no structures of the apo or binary NADH-bound forms are available for any mammalian LDH-A. Here, the apo structure of human LDH-A was solved at a resolution of 2.1 Å in space group *P*4_1_22. The active-site loop adopts an open conformation and the packing and crystallization conditions suggest that the crystal form is suitable for soaking experiments. The soaking potential was assessed with the cofactor NADH, which yielded a ligand-bound crystal structure in the absence of any inhibitors. The structures show that NADH binding induces small conformational changes in the active-site loop and an adjacent helix. A comparison with other eukaryotic apo LDH structures reveals the conservation of intra-loop interactions. The structures provide novel insight into cofactor binding and provide the foundation for soaking experiments with fragments and inhibitors.

## Abstract 2

A crystal structure of the human lactate dehydrogenase A apo form suitable for ligand soaking and a NADH binary-complex structure are presented.

## 1. Introduction   

**Figure 1:**
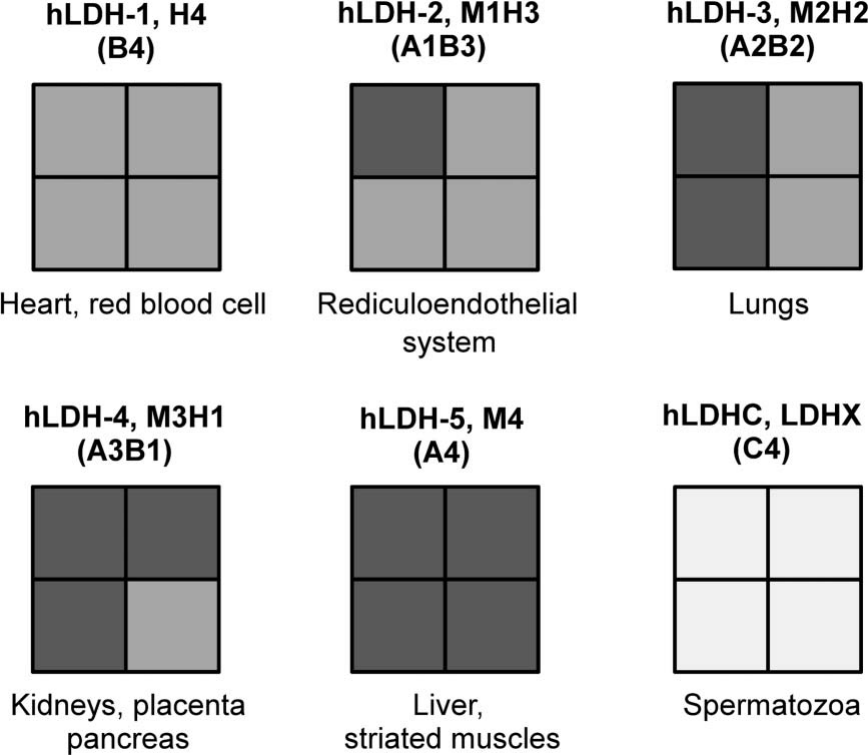
Schematic representation of the different forms of human LDH. The tetrameric LDH forms are shown using four squares, each representing a subunit of either LDH-A (dark grey), LDH-B (medium grey) or LDH-C (light grey). Alternative names and subunit compositions are shown at the top, and main tissue localizations are indicated at the bottom.

Lactate dehydrogenase (LDH) is a cytoplasmic enzyme that is present in essentially all major phyla. It serves as a catalyst for the NADH/NAD^+^-driven interconversion of pyruvate and lactate (Everse & Kaplan, 1973 ▶). In humans, lactate dehydrogenase (*h*LDH) occurs as homotetramers or heterotetramers predominantly comprising subunits encoded by the *LDH-A* (or *LDH-M*, muscle) and *LDH-B* (or *LDH-H*, heart) genes that assemble to form *h*LDH-1 (B_4_), *h*LDH-2 (AB_3_), *h*LDH-3 (A_2_B_2_), *h*LDH-4 (A_3_B) and *h*LDH-5 (A_4_). In addition, *h*LDHC_4_ is found in spermatozoa (Goldberg & Hawtrey, 1967 ▶). The different LDH forms are summarized in Fig. 1 ▶. The LDH-A and LDH-B subunits are similar in size and share 75% sequence identity, but have different catalytic properties; the A (or M) subunit preferentially converts pyruvate to lactate, while for the B (or H) form the converse is true (Koukourakis *et al.*, 2006 ▶). Mechanistically, LDH follows an ordered sequential mechanism whereby NADH binding to the cofactor-binding site precedes binding of pyruvate to the substrate-binding site; this is followed by a conformational change in which the active-site loop (residues 96–111 in *h*LDH) closes over the active site to provide a largely desolvated ternary complex. Molecular-dynamics calculations and temperature-jump relaxation spectroscopy have shown that in the open conformation, with no substrate or cofactor bound, the pocket is quite solvent-exposed; however, in the closed bound conformation the pocket is mostly buried (Pineda *et al.*, 2007 ▶; Qiu *et al.*, 2007 ▶). LDH-A (LDH-5) is well conserved across species for which amino-acid sequences are available, with typically more than 86% sequence identity observed in mammals. A number of mammalian LDH structures have been solved to date, including the enzymes from human, rat and rabbit (Read *et al.*, 2001 ▶; Ward *et al.*, 2012 ▶; Swiderek *et al.*, 2009 ▶; Dragovich *et al.*, 2013 ▶; Fauber *et al.*, 2013 ▶). However, all of these structures harbour the cofactor together with an inhibitor or alternatively are binary complexes with inhibitors, most of which span the cofactor-binding and substrate-binding pockets, and no apo structure of a mammalian LDH-A is available at present. The few eukaryotic LDH-A apo structures that have been solved so far are those from ice mackerel (71% sequence identity; PDB entry 2v65; Coquelle *et al.*, 2007 ▶) and spiny dogfish (79% sequence identity; PDB entry 6ldh; Abad-Zapatero *et al.*, 1987 ▶). An apo structure of human LDH-A is highly desirable as it could be used for soaking experiments with fragments and inhibitors for drug-discovery projects and may in addition reveal novel structural insights into human LDH-A.

Recently, LDH has attracted renewed interest owing to its prevalence in clinical tumours, where high expression often correlates with poor prognosis (Zhou *et al.*, 2010 ▶; Granchi *et al.*, 2011 ▶; Koukourakis *et al.*, 2003 ▶; Granchi & Minutolo, 2012 ▶). Serum and plasma *h*LDH-5 levels are used as tumour biomarkers (Koukourakis *et al.*, 2009 ▶). LDH levels are not necessarily correlated with nonspecific cellular damage, but rather overexpression is induced in malignant tumour phenotypes. Similarly, amplified expression of this gene is also found in several tumour cell lines, reflecting increased glycolysis arising from oxygen deprivation (Sheng *et al.*, 2009 ▶).

The provenance of *h*LDH-A in cancer metabolism and any potential structural changes observed in the human apo LDH structure, coupled with our interest in target-focused drug discovery (Moorhouse *et al.*, 2011 ▶), stimulated our search for a structure of the apo form of *h*LDH-A to assist in future fragment-based soaking experiments (Ward *et al.*, 2012 ▶; Kohlmann *et al.*, 2013 ▶). Here, we report the 2.1 Å resolution crystal structure of human LDH-A in the absence of any cofactor or inhibitor as well as the binary-complex structure with NADH.

## 2. Materials and methods   

### 2.1. Cloning and protein expression   

IMAGE clone 4096518 encoding *h*LDH-A was obtained from Source Bioscience, Cambridge, England. The gene was amplified by PCR using the forward primer 5′-GGAATTCCATATGGCAACTCTAAAGGATCAG-3′ and the reverse primer 5′-CCGCTCGAGAAATTGCAGCTCCTTTTGCATC-3′ and cloned into pET-26b using *Nde*I and *Xho*I restriction sites. The construct was verified by sequencing. For expression, the resulting plasmid was transformed into chemically competent *Escherichia coli* BL21 CP cells. Typically, six flasks containing 800 ml 2×YT medium supplemented with chloramphenicol and kanamycin were inoculated with 35 ml of overnight culture and incubated at 37°C with agitation (180 rev min^−1^). Cell growth was monitored by measuring the optical density at 600 nm until a value between 0.5 and 0.8 was reached, at which point isopropyl β-d-1-thiogalactopyranoside (IPTG) was added to a final concentration of 0.2 m*M* to induce the expression of recombinant *h*LDH-5 enzyme. 4 h after induction, cells were harvested by centrifugation at 4600*g* for 30 min. The final cell pellet was stored at −80°C.

### 2.2. Protein purification and activity assay   

Cell pellets were resuspended in 15–50 ml 200 m*M* KCl, 50 m*M* Tris–HCl, 20 m*M* imidazole at pH 8.0 and disrupted by sonication. Clarified lysate was loaded onto a nickel-immobilized affinity chromatography column and bound proteins were eluted with an imidazole gradient. Peak fractions containing *h*LDH-A protein were concentrated and further purified by gel filtration using a buffer consisting of 50 m*M* Tris–HCl, 150 m*M* NaCl at pH 7.5. Pure protein at a concentration of 20 mg ml^−1^ was aliquoted and stored at −80°C until further use. The specific activity for the recombinant enzyme was measured *via* UV spectrophotometry by monitoring the depletion of NADH at 340 nm in the presence of pyruvate.

### 2.3. Crystallization   

Crystals of full-length apo *h*LDH-A were grown using sitting-drop vapour diffusion at 19°C. Crystals were obtained by mixing equal volumes of a well solution consisting of 10% PEG 8000, 100 m*M* Na HEPES pH 7.5, 20% ethylene glycol, 10% acetonitrile with a protein solution comprising 8 mg ml^−1^ *h*LDH-5 in the gel-filtration buffer. Crystals were cryoprotected in a solution containing 90% well solution and 10% PEG 550 MME and cooled to 100 K in liquid nitrogen. For ligand soaking, compounds were dissolved in the cryoprotectant solution to a concentration of 1 m*M* and crystals were transferred into this solution, incubating for about 1 min prior to cryocooling.

### 2.4. Data collection, structure determination and refinement   

Diffraction data were collected from tetragonal crystals either solely cryoprotected or additionally previously soaked with NADH on beamline I04-1 at the Diamond Light Source (DLS) at 100 K and a wavelength of 0.9173 Å. Data were processed using *XDS* (Kabsch, 2010 ▶) and scaling was performed with *SCALA* from the *CCP*4 suite (Winn *et al.*, 2011 ▶). Data-collection and reduction statistics are summarized in Table 1 ▶. The crystals belonged to space group *P*4_1_22, with unit-cell parameters *a* = *b* = 84.7, *c* = 276.0 Å. The structure was solved by molecular replacement using *Phaser* (McCoy *et al.*, 2007 ▶) from the *CCP*4 suite (Winn *et al.*, 2011 ▶) with a monomer of *h*LDH with ligands removed as a search model (Read *et al.*, 2001 ▶). The structure contained two molecules in the asymmetric unit. After one round of rigid-body refinement with *PHENIX* (Adams *et al.*, 2010 ▶), manual rebuilding was performed in *Coot*, predominantly adjusting residues in the flexible-loop region (residues 99–110) and at the N-terminus. Subsequently, further rounds of restrained refinement were performed and waters were added and adjusted. The electron density was good throughout the entire model, which consisted of residues Ala1–Phe331. A sulfate or phosphate ion is potentially bound between the side chains of Arg168 and His192 with partial occupancy, but this was not modelled.

For the crystals soaked with NADH, data were collected on beamline I04-1 at DLS at 100 K. Rigid-body and positional refinement was carried out using *PHENIX* (Adams *et al.*, 2010 ▶) alternated with manual inspection and partial rebuilding in *Coot* (Emsley & Cowtan, 2004 ▶). The final structures were validated using *MolProbity* (Chen *et al.*, 2010 ▶). Refinement and geometric analysis statistics are summarized in Table 1 ▶. The atomic coordinates and structure factors have been deposited in the Protein Data Bank with accession codes 4l4r (apo structure) and 4l4s (NADH binary complex).

## 3. Results   

### 3.1. Recombinant human LDH-A preparation, crystallization and structure solution   

We cloned and expressed *h*LDH-A with a C-terminal His tag for easy purification purposes. Expression and subsequent purification produced highly pure protein with a typical yield of about 4 mg per litre of culture. The specific activity of the recombinant enzyme was measured by monitoring the depletion of NADH in the presence of pyruvate, and was calculated to be 163 ± 19 µmol min^−1^ mg^−1^ based on measurements in triplicate. In comparison, the activity of the commercially available enzyme (Lee Biosolutions) was 104 µmol min^−1^ mg^−1^.

We initially observed a crystallization hit using PEG 8000 and ethylene glycol as precipitant agents buffered with Na HEPES pH 7.5. The best of these crystals diffracted to 2.6 Å resolution and belonged to space group *P*2_1_2_1_2_1_, with unit-cell parameters *a* = 85.3, *b* = 141.2, *c* = 283.8 Å, which differed from the unit-cell parameters of the ternary NADH–oxamate crystal structure in the same space group reported previously (Read *et al.*, 2001 ▶). This crystal form contained two tetramers in the asymmetric unit, and inspection of the electron density revealed this to be an apo form of the enzyme (data not shown). Efforts were subsequently focused towards obtaining a higher resolution superior apo LDH-A crystal form for soaking experiments. Using additive screens, the addition of acetonitrile yielded fewer and larger crystals. Variation of the ethylene glycol concentration finally resulted in longer needle-like crystals with approximate dimensions of 20 × 300 × 20 µm. Several data sets from these crystals were obtained, the best of which extended to 2.1 Å resolution. These crystals belonged to a novel crystal form of hLDH-A in the tetragonal space group *P*4_1_22, with unit-cell parameters *a* = *b* = 84.7, *c* = 276.0 Å. Data-collection statistics are summarized in Table 1 ▶. The structure was solved by molecular replacement and two copies of LDH-A were found in the asymmetric unit, accounting for half of the tetramer, with a solvent content of about 64% (Matthews coefficient of 3.43 Å^3^ Da^−1^). The final model consisted of the entire protein sequence of 331 amino acids per monomer.

### 3.2. Characteristics of the apo LDH structure   

**Figure 2:**
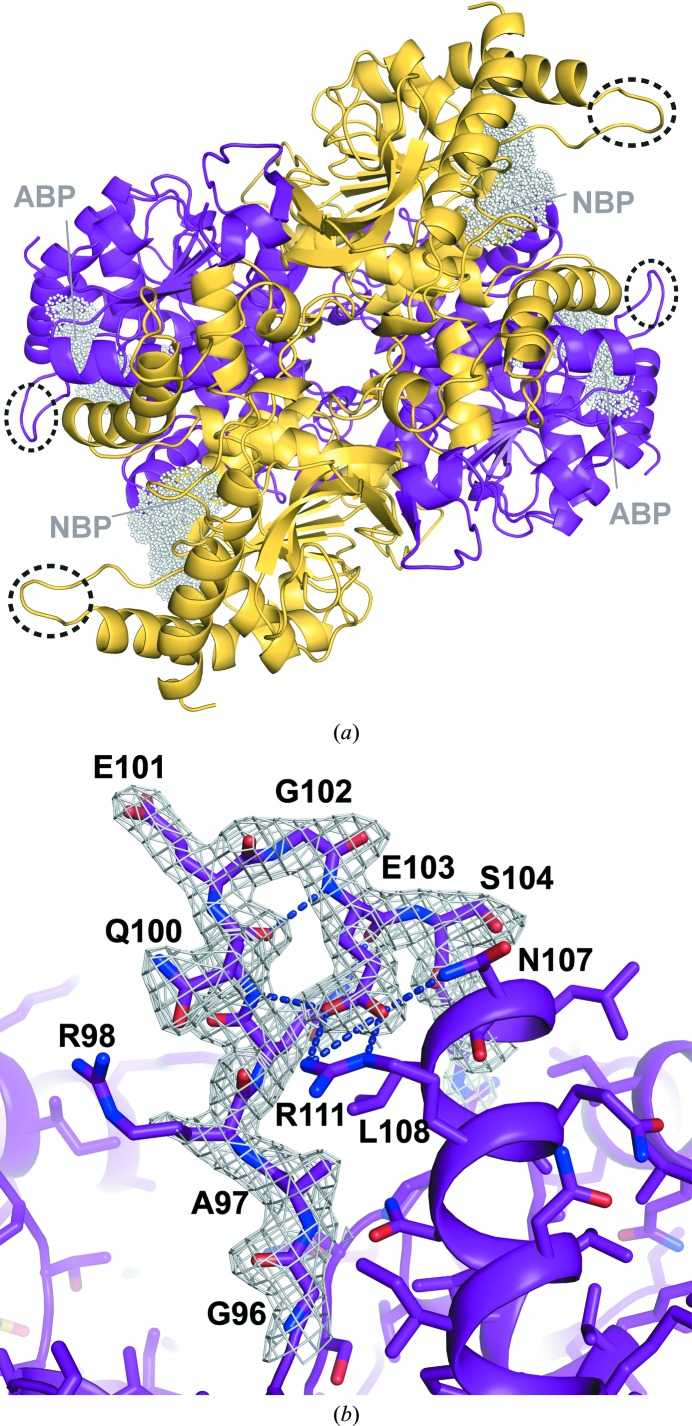
(*a*) Cartoon representation of the LDH-A apo-structure tetramer with crystallographically independent molecules depicted in purple and yellow, respectively. Active-site loops are circled and the location of the NADH-binding pocket is indicated by white spheres. NBP denotes the ‘nicotinamide moiety binding pocket’ and ABP denotes the ‘adenine moiety binding pocket’. (*b*) Close-up view of the σ_A_-weighted 2*F*_obs_ − *F*_calc_ electron-density map of the active-site loop contoured at the 1.1σ level (grey) with residues labelled and side chains shown as sticks depicted in purple.

The two LDH-A molecules in the asymmetric unit are almost identical and can be superimposed with an r.m.s.d. of 0.18 Å. *B*-factor analysis of the structure indicates a number of regions with increased flexibility, including the N-terminal tails and the active-site loop, in agreement with previous observations (Read *et al.*, 2001 ▶). The most striking feature of the human apo LDH structure relates to the conformation of the active-site loop comprising residues 99–110 (Fig. 2 ▶), which is often referred to as the substrate-specificity loop. Loop closure over the active site when substrate is bound is the rate-limiting step in the reduction of pyruvate (Gerstein & Chothia, 1991 ▶). In available binary complexes with NADH and apo structures of LDH from different species this loop is generally disordered. In the human apo structure reported here, the active-site loop is well ordered in the electron density and is observed in an ‘open’ conformation (Fig. 2 ▶ *b*). The active-site loops in the two molecules in the asymmetric unit are engaged in different packing interactions and accordingly show slightly different conformations. Nevertheless, equivalent intra-loop interactions are observed. Glu103 forms a salt-bridge interaction with Arg111 and this may contribute to the stabilization of this conformation of the type II β-turn. Furthermore, Glu103 also engages in a hydrogen-bonding interaction with the side chain of Asn107 and the main-chain amine of Gln100. Leu108 packs against the side chains of Gln99 and Ala97. In addition, this ordered conformation may be stabilized by crystal contacts with neighbouring monomers in the lattice, whereby two of these loop regions partly pack against each other. In the closed conformation of the ternary complex Gly96, Ala97, Arg98, Gln99 and Arg105 interact with both the pyruvate mimic oxamate and NADH. The density for the side chain of Arg105 is not well defined and indicates flexibility in the apo structure. In the main conformation observed, Arg105 forms a salt bridge with Glu191. Gln99 is well ordered and interacts with the main-chain carbonyl of Glu103, stabilizing the conformation of the active-site loop. In the NADH-binding pocket, density for the side chain of Arg98 is poorly defined in the structure, indicating flexibility in the apo form of the enzyme. Other active-site residues, including Arg168 and the catalytic His192 and Thr247, are well defined in the electron density.

### 3.3. The apo structure is amenable to ligand soaking   

**Figure 3:**
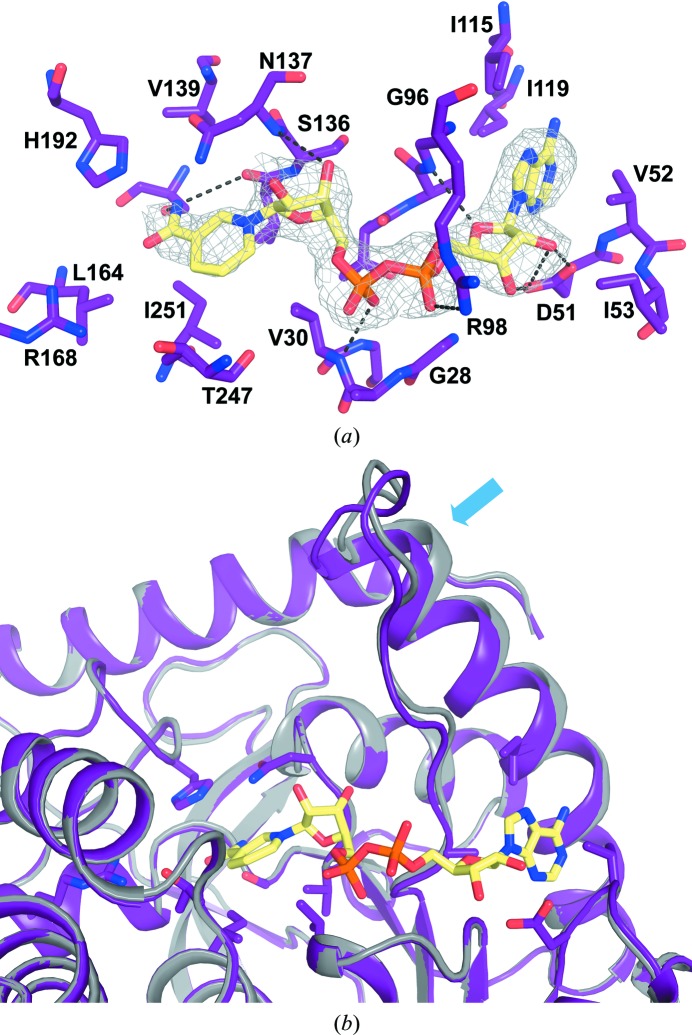
(*a*) σ_A_-weighted *F*_obs_ − *F*_calc_ electron-density map contoured at the 3σ level (grey) observed after soaking the apo LDH-A crystal form with NADH. The surrounding active-site region with residues lining the pocket shown as sticks is depicted in purple, with the modelled NADH depicted as yellow sticks. (*b*) Superposition of the crystal structures of the apo form (grey) and the NADH-bound binary complex (purple) of human LDH-A. The small-scale structural changes of the active-site loop and adjacent helix are indicated by an arrow.

In the crystal packing, large solvent channels are available to allow access to the active site. The high solvent content together with the neutral pH, low salt concentration and presence of relatively hydrophobic agents in the crystallization condition renders this crystal form highly suitable for soaking with small-molecule ligands. We investigated this possibility using the cofactor NADH as a proof-of-principle ligand and measured a data set from a crystal that had been soaked with NADH to 2.9 Å resolution. This experiment resulted in clearly identifiable additional electron density in the active site of *h*LDH-A, as shown in Fig. 3 ▶(*a*). The average *B* factors for NADH are similar to the *B* factors of surrounding residues, indicating full occupancy of the ligand. A superposition of the apo and NADH binary-complex structures focusing on the active-site region is shown in Fig. 3 ▶(*b*). The active-site loop remains in the open conformation after soaking, probably owing to its involvement in crystal contacts. In helix αD (the historical nomenclature is employed throughout the manuscript; Supplementary Fig. S1^1^) small-scale but significant movements of about 1.8 Å between residues Ile119 and Arg105 were observed after soaking with NADH, propagating a slight bending motion of the entire loop region until residue Ala105 (indicated by an arrow in Fig. 3 ▶ *b*). The intra-loop interactions such as the salt bridge between Arg111 and Glu103 are thereby maintained. The presence of NADH displaces at least seven water molecules, although a full analysis of the water-interaction network is hampered by the moderate resolution of the NADH binary-complex structure. In contrast to the apo structure, the side chain of Arg98 engages in contacts with the NADH phosphates and appears to be more ordered, with the density being better resolved. In addition to the active-site loop and helix αD movements, a number of residues display rotamer changes upon binding of NADH, including residues Val25, Ile53, Glu54, Lys56, Ile115, Asn137, Ser160, Leu164, Thr247, Ile251, Ile325, Glu328 and Gln330.

### 3.4. Comparison between the apo and binary and ternary complex structures   

**Figure 4:**
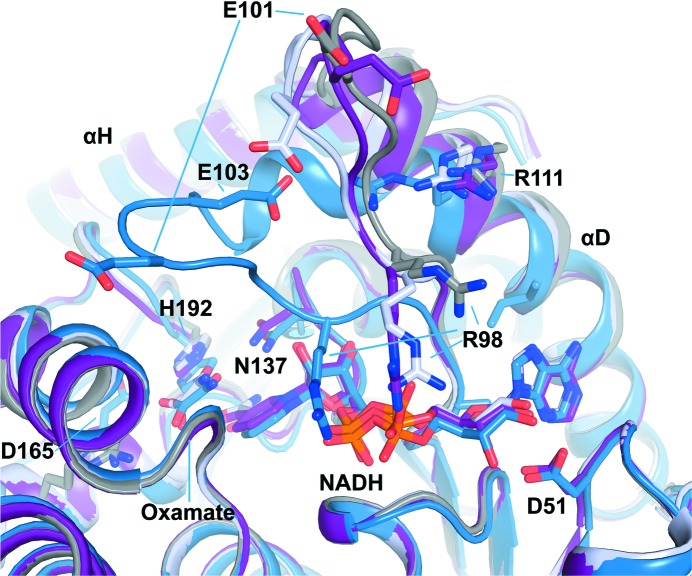
Superposition of the human LDH-A apo form (grey) and NADH binary complex (purple) reported here with the ternary complex bound to NADH and the competitive inhibitor oxamate (two different monomers shown in blue and light blue, respectively; PDB entry 1i10; Read *et al.*, 2001 ▶), highlighting the conformational changes upon cofactor and substrate binding, with key residues shown in stick representation.

The apo and binary NADH-bound structures presented here in combination with the previously determined ternary complex (PDB entry 1i10; Read *et al.*, 2001 ▶) allow a direct comparison between the apo and NADH-bound binary and ternary complex structures of human lactate dehydrogenase A. A monomer from the apo structure can be superimposed with monomers from the oxamate and NADH-bound structure in a different space group with an r.m.s.d. of 0.5 Å over 300 residues (excluding residues from the active-site loop and N-terminus). This superposition highlights the large-scale conformational changes observed in the loop region (Fig. 4 ▶). In the closed conformation of the ternary complex, residues Gln99, Arg105, Asn137, Arg168, His192 and Thr247 are engaged in hydrogen-bonding interactions with oxamate, while Leu164 and Ala237 are engaged in hydrophobic contacts. The side chain of Arg168 interacts with the carboxylate group of the ligand. A protonated His192 is the catalytically important proton donor in the pyruvate-to-lactate conversion and together with Arg105 is crucial for pyruvate binding. In the binary-complex and apo structures, Arg105 is exposed to bulk solvent and interacts with Glu191. The guanidinium moiety undergoes movements of over 10 Å from the surface to a buried position upon substrate binding. Glu101 at the tip of the loop moves over 8–11 Å between the open and closed forms in both independent molecules of the asymmetric unit. In one monomer of the ternary NADH/oxamate structure the active site is substantially more open (PDB entry 1i10 chain *D*; Read *et al.*, 2001 ▶), possibly capturing an intermediate conformational state before loop closure. Hence, together with the structures presented here, four forms of human LDH-A can be compared with the caveat that the mobile loop is involved in crystal contacts. The two NADH molecules in our binary complex and in the half-open monomer of the ternary complex superimpose well, whereas in the closed form of the ternary complex NADH appears to be shifted deeper into the binding pocket. Loop closure is associated with additional small movements in the αD helix (Ala95–Ile119) and the C-terminal αH helix (Fig. 4 ▶). In addition, local differences are observed in a number of side-chain conformations, including Ile53, Lys56, Asn137, Arg168, Ser236, Ala237, Tyr238 and Ile241. The catalytic residue His192 slightly shifts towards the substrate mimic by about 1.1 Å only in the closed conformation of the active-site loop. This coincides with a shift of Asn137, while the conformation of Asp165 remains virtually unchanged.

### 3.5. Comparison with other eukaryotic LDH-A structures   

**Figure 5:**
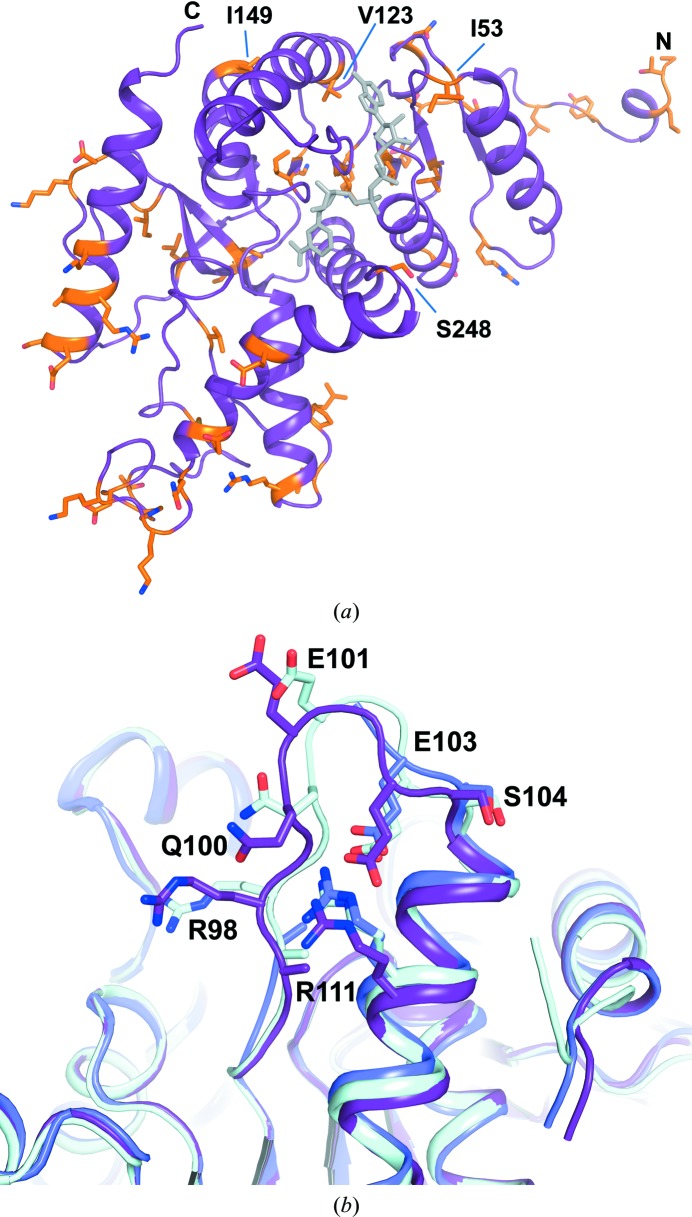
(*a*) Cartoon representation of a monomer of the human LDH-A binary complex (purple) with NADH (grey) with positions of sequence variation among mammalian LDH-A enzymes shown as sticks and coloured in orange. Selected residues in close proximity to the active site are labelled. (*b*) Superposition of the human LDH-A apo form (purple) with LDH-A apo structures from spiny dogfish (cyan; PDB entry 6ldh; 0.45 Å r.m.s.d., 79% sequence identity; Abad-Zapatero *et al.*, 1987 ▶) and ice mackerel (light blue; PDB entry 2v65; 0.6 Å r.m.s.d., 71% sequence identity; Coquelle *et al.*, 2007 ▶).

Whereas structures of ternary complexes and binary complexes with inhibitors of mammalian LDH-A orthologues have been determined, no other apo structure is available for comparison. There are some residues that are affected by small structural changes upon NADH and substrate binding and are not absolutely conserved among mammals, including residues Ile53 and Ser248 (Fig. 5 ▶ *a*, Supplementary Fig. S1). Residues in the active-site loop and α-helix D are absolutely conserved among mammals, with the exception of Val123. Additionally, Ile149, which backs onto this α-helix, is not absolutely conserved. Residues 12–19 display high *B* factors, but the positions of the active-site residues are almost identical in these ternary inhibitor structures. The closest LDH-A apo structures available at present are from *Squalus acanthias* (spiny dogfish; PDB entry 6ldh; 79.9% sequence identity; Abad-Zapatero *et al.*, 1987 ▶) and from *Champsocephalus gunnari* (ice mackerel; PDB entry 2v65; 71.5% sequence identity; Coquelle *et al.*, 2007 ▶). The sequence in the active-site loop is absolutely conserved, but a much higher degree of sequence variation occurs in other parts of the enzyme compared with mammalian LDH-As. Despite different crystal-packing arrangements in the human and dogfish apo structures, the active-site loop regions nevertheless adopt similar conformations, with a local r.m.s.d. of 0.4 Å over the C^α^ atoms of 20 residues. Interestingly, the salt bridge between Glu103 and Arg115 (Fig. 5 ▶ *b*) is also conserved among these three apo crystal structures, even though residues 101–105 are disordered in the structure from ice mackerel. This suggests that despite its flexible nature, the crystal structures may capture a preferred conformation of the active-site loop in apo forms of this enzyme.

## 4. Discussion   

Lactate dehydrogenase has been studied for many decades (Markert & Møller, 1959 ▶) as it is abundant in tissue cells and is a marker for cell damage. Its position in the glycolytic cascade renders it a viable anticancer target, which has initiated a renaissance of research into this important enzyme. In this study, we close an important gap by presenting the apo and binary NADH-bound structures of human LDH-A. Moreover, the structure of the apo form together with our soaking experiment with NADH demonstrates that the active site is accessible to small-molecule ligands in this crystal form. Recently, ligand-bound crystal structures of rat LDH have been reported (Ward *et al.*, 2012 ▶), and rabbit LDH-A has also been used as model system for inhibitor design (Kohlmann *et al.*, 2013 ▶). The rat LDH-A crystals belonged to space group *P*2_1_; they were grown in high-salt conditions with four molecules in the asymmetric unit and showed a closed conformation of the active site. LDH-A crystals grown in the presence of AMP from rabbit that belonged to space group *P*2_1_ with two tetramers in the asymmetric unit and a disordered active-site loop have also been used for soaking experiments.

Although both rat and rabbit LDH-A are highly homologous and the active-site residues are largely conserved compared with human LDH-A, there are nevertheless small differences in the active-site region (Fig. 5 ▶ *a*, Supplementary Fig. S1). Single amino-acid substitutions can have long-range effects on protein structure, dynamics and ligand interactions that can potentially have consequences for using these systems as surrogates for drug design. In our crystal form the loop is observed in an open conformation, readily allowing access to the binding pocket, and is stabilized through crystal contacts. Our results show that LDH-A is able to bind NADH in this open conformation. As loop closure is restricted, ligands interacting with the pyruvate and/or nicotinamide binding pocket may display a decreased affinity. However, substrate mimics can bind in this conformation, as observed in monomers with an open active-site loop in human and rabbit ternary-complex structures (Swiderek *et al.*, 2009 ▶; Read *et al.*, 2001 ▶). This demonstrates that loop closure is not a prerequisite for substrate-mimic binding. It has been suggested that multiple binding pathways for the substrate are likely (Qiu *et al.*, 2007 ▶). The apo structure described here will provide an important platform for drug design and the understanding of this key enzyme in glycolysis.

Our ligand-bound structure reveals NADH bound in an almost identical fashion to that seen previously in the ternary complex in the open conformation, implying that the presence or absence of oxamate does not have a significant impact on the NADH conformation. The binding of NADH only triggers small-scale local changes, indicating that the mobility of the active-site loop is hardly restricted by the presence of bound NADH. This is also supported by *B*-factor analysis, which shows that the average *B* factors of the loop residues are comparable between the NADH-bound and apo structures. Rather, the NADH position is slightly affected by loop closure, which coincides with a slight repositioning of the catalytic residues, including His192. This is in line with this being the rate-limiting step in the catalytic cycle. Although the loop has often been observed to be disordered in the open conformation, our data suggest that some intra-loop inter­actions are conserved, indicating that preferred conformations of the apo and NADH-bound active-site loop exist. Some of these interactions are maintained after loop closure, such as the salt bridge between Glu103 and Arg111.

Taken together, our structural study of human LDH-A in the apo form and in complex with NADH adds to our understanding of the conformational changes during catalysis and the conservation of intra-loop interactions across higher eukaryotes and will provide a valuable tool for inhibitor design.

## Supplementary Material

PDB reference: human LDH-A, apo, 4l4r

PDB reference: NADH binary complex, 4l4s

Supplementary Figure 1.. DOI: 10.1107/S1399004714005422/mn5051sup1.pdf

## Figures and Tables

**Table 1:** Data-collection and refinement statistics Values in parentheses are for the last shell.

	Apo	NADH-bound
Data collection		
Wavelength (Å)	0.9173	0.9173
Space group	*P*4_1_22	*P*4_1_22
Unit-cell parameters (Å)	*a* = *b* = 84.7, *c* = 276.0	*a* = *b* = 84.3, *c* = 276.7
*d*_min_ (Å)	2.1	2.9
Completeness (%)	94.3 (75.0)	99.8 (99.9)
Total reflections	368718	131336
Unique reflections	56269	23011
Multiplicity	6.6 (5.7)	5.7 (5.6)
〈*I*/σ(*I*)〉	14.9 (3.9)	7.7 (2.5)
Wilson plot *B* factor (Å^2^)	29.0	44.0
*R*_merge_ †	0.077 (0.480)	0.203 (0.770)
CC_1/2_ ‡	0.998 (0.863)	0.988 (0.902)
*R*_p.i.m._ §	0.032 (0.217)	0.092 (0.350)
Refinement		
*R* factor¶ (%)	16.4	18.5
*R*_free_ †† (%)	18.2	24.6
No. of residues (atoms)	662 (5160)	662 (5160)
No. of ligands (atoms)	—	2 (86)
No. of waters	300	37
R.m.s.d., bond lengths (Å)	0.009	0.0013
R.m.s.d., bond angles (°)	1.2	1.5
Average *B* factors (Å^2^)		
Protein	36.4	39.1
NADH	—	37.0
Waters	43.6	23.5
Ramachandran plot		
Preferred regions (%)	98.2	97.6
Allowed regions (%)	1.8	2.4

† *R*_merge_ = 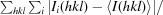 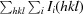.

‡ CC_1/2_ is the Pearson correlation coefficient between random half-data sets.

§ *R*_p.i.m._ = 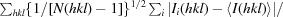 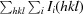, where 〈*I*(*hkl*)〉 is the average intensity and *I_i_*(*hkl*) is the *i*th measurement of reflection *hkl*.

¶ *R* factor = 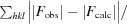 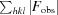.

†† *R*_free_ corresponds to the *R* factor based on 5.1% of the data which were excluded from refinement.

## Footnotes

1 Supporting information has been deposited in the IUCr electronic archive (Reference: MN5051).
