# Supplementary material for: Structural characterization of the apo form and NADH binary complex of human lactate dehydrogenase
Source: Acta Crystallogr D Biol Crystallogr. 2014 Apr 30;70(Pt 5):1484–90. doi: 10.1107/S1399004714005422 (PMC4014127; doi:10.1107/S1399004714005422)
Supplement: Supplementary file 1 [file d-70-01484-sup1.pdf]

Supplementary Figure 1

```
                     α1 (A)                β1 (A)      α2 (B)          β2 (B)    α3 (C)
                     HHHHH                EEEEEEE HHHHHHHHHHHH        EEEEE    HHHHHH
                                               ●●●                        ♦♦
LDHA_HUMAN    MATLKDQLIYNLLKEEQTPQNKITVVGVGAVGMACAISILMKDLADELALVDVIEDKLKG
LDHA_BOVIN    MATLKDQLIQNLLKEEHVPQNKITIVGVGAVGMACAISILMKDLADEVALVDVMEDKLKG
LDHA_PIG      MATLKDQLIHNLLKEEHVPHNKITVVGVGAVGMACAISILMKELADEIALVDVMEDKLKG
LDHA_RABIT    MAALKDQLIHNLLKEEHVPQNKITVVGVGAVGMACAISILMKDLADELALVDVMEDKLKG
LDHA_MOUSE    MATLKDQLIVNLLKEEQAPQNKITVVGVGAVGMACAISILMKDLADELALVDVMEDKLKG
LDHA_RAT      MAALKDQLIVNLLKEEQVPQNKITVVGVGAVGMACAISILMKDLADELALVDVIEDKLKG
LDHA_MONDO    MGTVKDQLILNVLKEEQTPHNKITVVGVGAVGMACAISILMKDLADELALVDVIEDKLKG
              *.::***** *:****:.*:****:****************:****:*****:******

                          β3 (C)        β4 (D)             α4 (D)
              HHHHHHH GGG    EEEE   GGGG   EEEE      TT   HHHHHHHHHHHHHHH
                                     ♦          ●♦♦ ●●    ●          ♦   ♦
LDHA_HUMAN    EMMDLQHGSLFLRTPKIVSGKDYNVTANSKLVIITAGARQQEGESRLNLVQRNVNIFKFI
LDHA_BOVIN    EMMDLQHGSLFLRTPKIVSGKDYNVTANSKLVIITAGARQQEGESRLNLVQRNVNIFKFI
LDHA_PIG      EMMDLQHGSLFLRTPKIVSGKDYNVTANSRLVVITAGARQQEGESRLNLVQRNVNIFKFI
LDHA_RABIT    EMMDLQHGSLFLRTPKIVSGKDYSVTANSKLVIITAGARQQEGESRLNLVQRNVNIFKFI
LDHA_MOUSE    EMMDLQHGSLFLKTPKIVSSKDYCVTANSKLVIITAGARQQEGESRLNLVQRNVNIFKFI
LDHA_RAT      EMMDLQHGSLFLKTPKIVSSKDYSVTANSKLVIITAGARQQEGESRLNLVQRNVNIFKFI
LDHA_MONDO    EMMDLQHGSLFLKTPKIVSSKDYAVTANSKLVVITAGARQQEGESRLNLVQRNVNIFKFI
              ************:******.*** *****:**:***************************

               α4 (E)    β5 (E)     α5 (1F)         β6 (F)        α6 (2F)
              HHHHHHH    EEEE    HHHHHHHHHHHH    GGGEEE    HHHHHHHHHHHHHHH
                             ●●●                        ●    ●  ●
LDHA_HUMAN    IPNVVKYSPNCKLLIVSNPVDILTYVAWKISGFPKNRVIGSGCNLDSARFRYLMGERLGV
LDHA_BOVIN    IPNIVKYSPNCKLLVVSNPVDILTYVAWKISGFPKNRVIGSGCNLDSARFRYLMGERLGV
LDHA_PIG      IPNIVKYSPNCKLLVVSNPVDILTYVAWKISGFPKNRVIGSGCNLDSARFRYLMGERLGV
LDHA_RABIT    IPNVVKYSPHCKLLVVSNPVDILTYVAWKISGFPKNRVIGSGCNLDSARFRYLMGERLGV
LDHA_MOUSE    IPNIVKYSPHCKLLIVSNPVDILTYVAWKISGFPKNRVIGSGCNLDSARFRYLMGERLGV
LDHA_RAT      IPNVVKYSPQCKLLIVSNPVDILTYVAWKISGFPKNRVIGSGCNLDSARFRYLMGERLGV
LDHA_MONDO    IPNIVKYSPNCKLLVVSNPVDILTYVAWKLSGFPKNRVIGSGCNLDSARFRYLMGEKLGI
              ***:*****:****:**************:**************************:**:

                     β7 (G)     β8 (H)    β9 (J) β10  α7                α8 (1G) (2G)
               GGG     EE     TT EE GGG EETTEEHHHH        TT  TT    HHHHHHHHHHHHH
                          ●
LDHA_HUMAN    HPLSCHGWVLGEHGDSSVPVWSGMNVAGVSLKTLHPDLGTDKDKEQWKEVHKQVVESAYE
LDHA_BOVIN    HPLSCHGWILGEHGDSSVPVWSGVNVAGVSLKNLHPELGTDADKEQWKAVHKQVVDSAYE
LDHA_PIG      HPLSCHGWILGEHGDSSVPVWSGVNVAGVSLKNLHPELGTDADKEHWKAVHKQVVDSAYE
LDHA_RABIT    HALSCHGWILGEHGDSSVPVWSGMNVAGVSLKTLHPELGTDADKEQWKQVHKQVVDSAYE
LDHA_MOUSE    HALSCHGWVLGEHGDSSVPVWSGVNVAGVSLKSLNPELGTDADKEQWKEVHKQVVDSAYE
LDHA_RAT      HPLSCHGWVLGEHGDSSVPVWSGVNVAGVSLKSLNPQLGTDADKEQWKDVHKQVVDSAYE
LDHA_MONDO    HSSSCHGWILGEHGDSSVPVWSGVNVAGVSLKSLHPALGTDSDSEQWKDVHKQVVESAYE
              *. *****:**************:********.*:* **** *.*:** ******:****

                         α9 (3G)          β11 (K)               β12 (L)
              HHHHH    HHHHHHHHHHHHHHH    EEEEEEEE TT TT    EEEEEEEEETTEE
                      ●   ●
LDHA_HUMAN    VIKLKGYTSWAIGLSVADLAESIMKNLRRVHPVSTMIKGLYGIKDDVFLSVPCILGQNGI
LDHA_BOVIN    VIKLKGYTSWAIGLSVADLAESIMKNLRRVHPISTMIKGLYGIKEDVFLSVPCILGQNGI
LDHA_PIG      VIKLKGYTSWAIGLSVADLAESIMKNLRRVHPISTMIKGLYGIKEDVFLSVPCILGQNGI
LDHA_RABIT    VIKLKGYTTWAIGLSVADLAESIMKNLRRVHPISTMLKGLYGIKEDVFLSVPCVLGQNGI
LDHA_MOUSE    VIKLKGYTSWAIGLSVADLAESIMKNLRRVHPISTMIKGLYGINEDVFLSVPCILGQNGI
LDHA_RAT      VIKLKGYTSWAIGLSVADLAESIMKNLRRVHPISTMIKGLYGIKEDVFLSVPCILGQNGI
LDHA_MONDO    VIKLKGYTSWAIGLSVADLAESIMKNLRRVHPISTMIKGLYGINEDVFLSVPCILGQNGI
              ********:***********************:***:******::*********:******

              β13 (M)      α10 (H)
              EEEE      HHHHHHHHHHHHHHHHHHH

LDHA_HUMAN    SDLVKVTLTSEEEARLKKSADTLWGIQKELQF
LDHA_BOVIN    SDVVKVTLTHEEEACLKKSADTLWGIQKELQF
LDHA_PIG      SDVVKVTLTPEEEAHLKKSADTLWGIQKELQF
LDHA_RABIT    SDVVKVTLTSEEEAHLKKSADTLWGIQKELQF
LDHA_MOUSE    SDVVKVTLTPEEEARLKKSADTLWGIQKELQF
LDHA_RAT      SDVVKVTLTPDEEARLKKSADTLWGIQKELQF
LDHA_MONDO    SDVVKVTLTTEEESRLKQSADTLWGIQKELQF
              **:****** :**: **:**************
```

Multiple sequence alignment of human (UniProtKB accession code: P00338), bovine (UniProtKB accession code: P19858; 93.7% sequence identity), porcine (UniProtKB accession code: P00339; 92.8% sequence identity), rabbit (UniProtKB accession code: P13491; 93.7% sequence identity), murine (UniProtKB accession code: P06151, 93.9% sequence identity), rat (UniProtKB accession code: P04642; 94.3% sequence identity) and opposum (UniProtKB accession code: Q9XT87; 90.9% sequence identity) LDH-A. The active site loop is highlighted in yellow and the catalytic residue His193 is highlighted in orange. Other active site pocket residues are marked with a rhombus or dot depending on their proximity to the adenine or oxamate/nicotinamide moieties, respectively. Secondary structure elements are shown above the sequences whereby E denotes for β-strand, H for α-helix, G for 3_{10} helix and T for β-turn. A sequential and the historical (in brackets) nomenclature are shown for secondary structure elements.
